# Rab GTPase Prenylation Hierarchy and Its Potential Role in Choroideremia Disease

**DOI:** 10.1371/journal.pone.0081758

**Published:** 2013-12-16

**Authors:** Monika Köhnke, Christine Delon, Marcus L. Hastie, Uyen T. T. Nguyen, Yao-Wen Wu, Herbert Waldmann, Roger S. Goody, Jeffrey J. Gorman, Kirill Alexandrov

**Affiliations:** 1 Department of Cell and Molecular Biology, Institute for Molecular Bioscience, The University of Queensland, St Lucia, Queensland, Australia; 2 Queensland Berghofer Medical Research Institute, PO Royal Brisbane Hospital, Herston, Queensland, Australia; 3 Department of Chemical Biology, Max-Planck-Institute for Molecular Physiology, Dortmund, Germany; 4 Department of Physical Biochemistry, Max-Planck-Institute for Molecular Physiology, Dortmund, Germany; BioScience Project, United States of America

## Abstract

Protein prenylation is a widespread post-translational modification in eukaryotes that plays a crucial role in membrane targeting and signal transduction. RabGTPases is the largest group of post-translationally C-terminally geranylgeranylated. All Rabs are processed by Rab geranylgeranyl-transferase and Rab escort protein (REP). Human genetic defects resulting in the loss one of two REP isoforms REP-1, lead to underprenylation of RabGTPases that manifests in retinal degradation and blindness known as choroideremia. In this study we used a combination of microinjections and chemo-enzymatic tagging to establish whether Rab GTPases are prenylated and delivered to their target cellular membranes with the same rate. We demonstrate that although all tested Rab GTPases display the same rate of membrane delivery, the extent of Rab prenylation in 5 hour time window vary by more than an order of magnitude. We found that Rab27a, Rab27b, Rab38 and Rab42 display the slowest prenylation *in vivo* and in the cell. Our work points to possible contribution of Rab38 to the emergence of choroideremia in addition to Rab27a and Rab27b.

## Introduction

Post-translational modifications affect a large fraction of mammalian proteins and expand the complexity of the proteome. The mammalian Rab GTPase family, the largest of the Ras-like super-family of GTPases with over 60 members requires posttranslational lipidation for their activity. This modification enables them to function as regulators of numerous membrane trafficking steps, including vesicle budding, targeting, fusion and motility [1]. Rabs are post-translationally modified by the attachment of one or in most cases two geranylgeranyl moieties to the C-terminus by Rab geranylgeranyl-transferase (RabGGTase). Unlike the other prenyltransferases, farnesyl-transferase (FTase) and geranylgeranyl-transferase I (GGTaseI), RabGGTase does not rely on a consensus sequence such as the CAAX box, but instead has delegated substrate recognition to a third protein: Rab escort protein (REP) (for a review see Nguyen, Goody, and Alexandrov, 2010) [2,3]. The newly synthesized, unprenylated Rab is bound by REP and presented to RabGGTase. REP stays bound to Rab after attachment of hydrophobic geranylgeranyl groups, enabling the Rab to remain in solution until it is delivered to the appropriate membrane [4]. This delegation of substrate recognition has enabled the expansion of the Rab family and has allowed the C-terminus of Rabs to become hyper-variable [5].

Mammalian cells express two ubiquitous REP isoforms, REP1 and REP2, that share 75% amino acid identity and 90% amino acid similarity [6,7]. Loss-of-function mutations of REP1 in humans cause choroideremia, an X-linked recessive disease, which leads to the degeneration of photoreceptors, retinal pigment epithelium and the choroid in the eye and eventually results in blindness by middle age. It has been shown that choroideremic cells accumulate unprenylated Rab27a, but it is unclear if this is the only Rab that is affected by underprenylation [8,9]. It was initially assumed that Rab27a prenylation was dependent on REP1 and that Rab27a could not be prenylated by REP2. However, further work showed that, though Rab27a is prenylated less efficiently by REP2 than by REP1 (a fourfold difference), this difference is also seen with other Rabs e.g. Rab1a (>2 fold) and Rab7 (>6 fold) [4]. It was noted that the dissociation constant for Rab27a was significantly higher for both REPs than it was for Rab7a or Rab1a [4,9] and *in vitro* studies showed that when REP activity was limited, equimolar Rab7 could out-compete Rab27a prenylation [4]. This suggests that in the cell, where Rabs are competing with each other for binding to the prenylation complex, a Rab prenylation hierarchy might exist. This could lead to an underprenylation of Rabs with a low affinity for the prenylation machinery or slow turnover rate, under conditions where the prenylation activity is compromised.

In order to further elucidate the *in vivo* mechanism of Rab processing and the molecular basis of choroideremia, we present several lines of evidence that Rabs may have different prenylation rates *in vivo*, which is independent of the membrane delivery itself. We obtained a first snapshot of prenylation hierarchy of Rabs, identifying Rabs that are more vulnerable to a general reduction in prenylation capacity than others and in addition to Rab27a might be underprenylated in choroideremia patients. 

## Results

### Rab GTPases display different rates of *in vivo* prenylation

**Figure 1:**
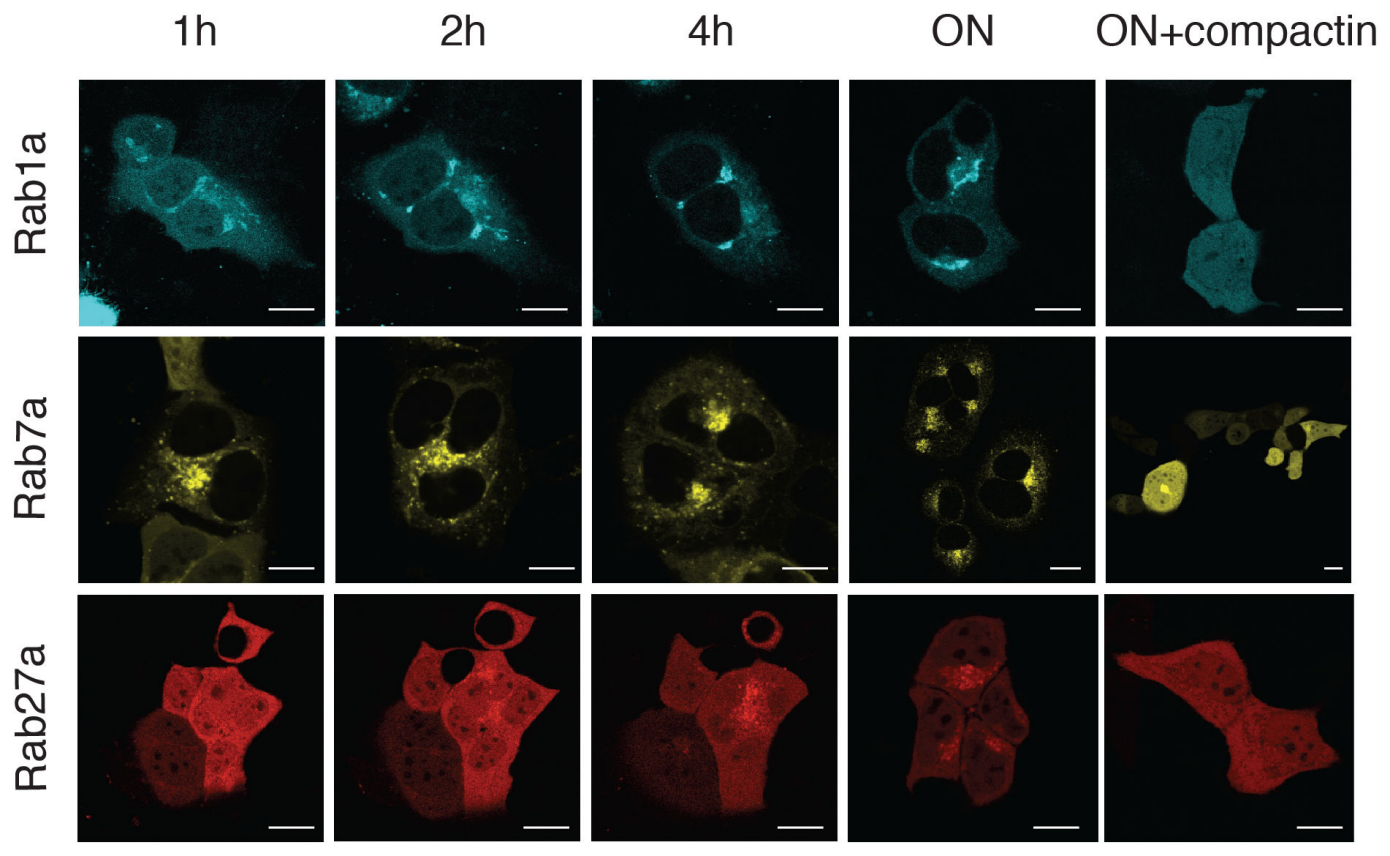
Rates of *in* *vivo* prenylation and localization for Rab1a, Rab7a and Rab27a. Unprenylated, fluorescently-tagged recombinant CFP-Rab1a (upper panel), YFP-Rab7a (middle panel) and Cherry-Rab27a (lower panel) were microinjected into A431 cells. Cells were imaged over 22h and cellular localization of the injected Rab proteins detected by their fluorescent tags. The timepoints after microinjection are displayed above the images in hours or overnight (ON). Cells were pre-incubated with compactin where stated. Scale bars represent 20 µm.

Interactions of the Rab prenylation machinery and differences in Rab prenylation rates have been studied extensively *in vitro*. As mentioned in the introduction, affinities of Rabs for REP molecules can differ by more than two orders of magnitude. In the situation where all substrates are present simultaneously this may give rise to competition among Rabs. In order to see whether the *in vitro* measured K_d_ values would correlate with *in vivo* prenylation kinetics, we microinjected unprenylated recombinant CFP-Rab1a, YFP-Rab7a and Cherry-Rab27a into A431 cells. Delivery of unprenylated GTPases to the cells via microinjection is well established method for their analysis [10]. The proteins were initially distributed through the cytoplasm and leaked into the nucleus. Over time the proteins localized to their target cell membranes, reflecting their prenylation. Changes of membrane associated fluorescence versus total cellular fluorescence provided us with an estimate of Rab association rates with the membrane for each Rab construct. YFP-Rab7a, which has the highest affinity for both REPs displayed the fastest membrane localization to its target organelles, late endosomes and lysosomes, reaching completion between 2 and 4h (Figure 1 middle panel). CFP-Rab1a, which displays an intermediate K_d_ values localized to its target membrane, the endoplasmic reticulum, on a somewhat longer timescale with complete localization requiring about 4h (Figure 1 upper panel). In contrast, Cherry-Rab27a, with a significantly greater K_d_ than Rab1a and Rab7a, associated with secretory granules as its target membrane, approximately an order of magnitude more slowly than YFP-Rab7a, showing only minimal localization after 4h and incomplete localization even after 22h (Figure 1 lower panel). Treatment of the microinjected cells with compactin, an inhibitor of the mevalonate pathway that reduces the endogenous pool of phosphoisoprenoids, prevented the localization of Rabs to the membrane in all cases (Figure 1). While precise quantification of protein localization rates to different compartments is difficult due to the differences in morphology of the target organelles, the large differences in time required for localization of individual Rabs is likely to reflect differences in efficiencies of the underlying processes. 

**Figure 2:**
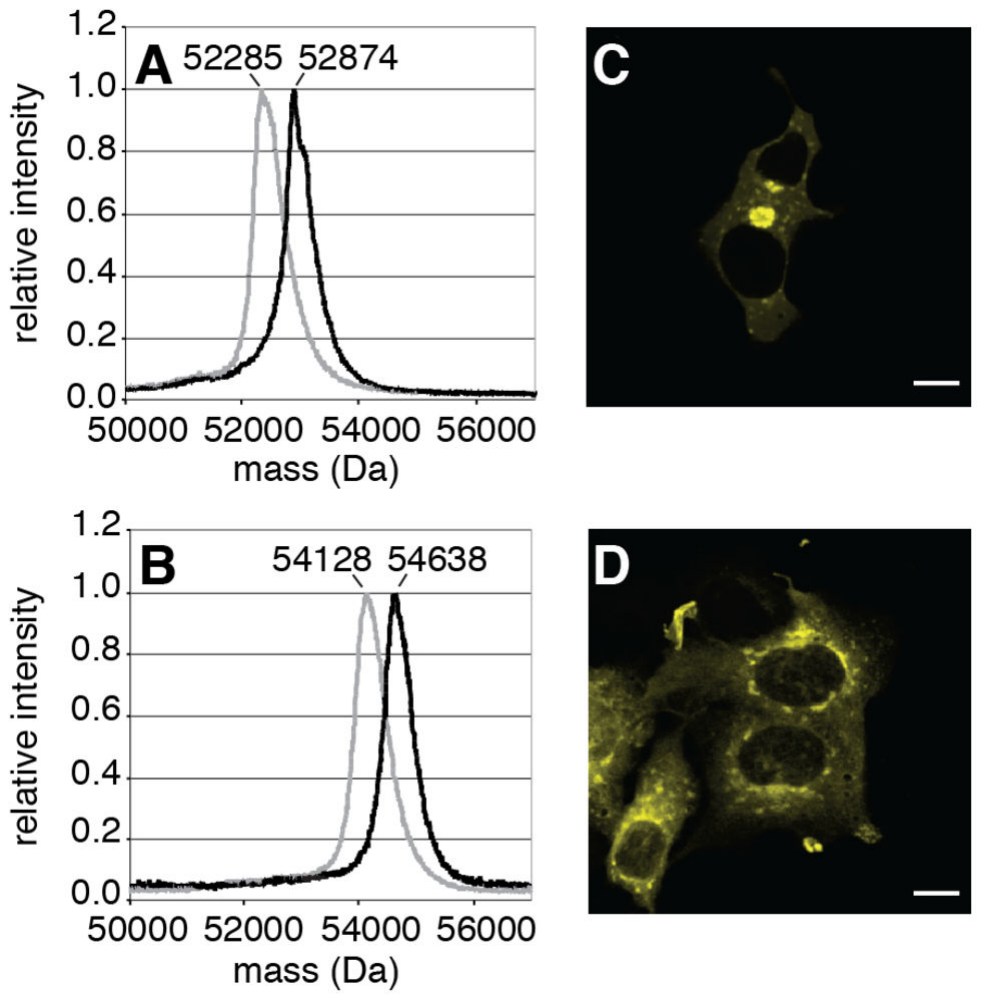
Localization is an indicator of prenylation. MALDI mass spectrometry comparison of unmodified (gray) and *in* *vitro* prenylated (black) Citrine-Rab7 (A) and Citrine-Rab27a (B). Average protein masses are indicated at the peaks. Prenylated Citrine-Rab7:REP1 (C) and prenylated Citrine-Rab27a:REP1 (D) were microinjected into compactin treated A431 cells. Membrane targeting of Rab proteins was detected by imaging the cells 20 min post-injection. Scale bars represent 10 µm.

Since the localization of Rab proteins to the membrane involves at least two steps, i.e. prenylation and membrane delivery, we wanted to determine which of these two steps dominates the kinetics of Rab association with the membrane. Therefore, we used a previously described procedure for *in vitro* production of prenylated Citrine-Rab7a:REP1 and Citrine-Rab27a:REP1 complexes [11] (Figure 2A-B). The protein complexes were microinjected into A431 cells, which were pre-incubated with compactin. Both Citrine-Rab7a and Citrine-Rab27a localized to their target membranes nearly completely in 20 min (Figure 2C-D). Since membrane delivery of prenylated Rab proteins is not rate limiting, we conclude that differences in localization kinetics may reflect differences in *in vivo* rates of prenylation in the cell.

The different rates of Rab prenylation in the cell would suggest a potential prenylation hierarchy with Rab27a being at a less preferable position. Rab7a that would be situated higher in this hierarchy, in keeping with its lower K_d_ for REPs, and can potentially out-compete certain other endogenous, newly synthesized Rabs, becoming relatively quickly prenylated and delivered to membranes. Under the same circumstances, excess Rab27a, much lower on the prenylation hierarchy due to its higher K_d_ for both REPs, would be out-competed by endogenous Rabs and therefore Rab27a prenylation and subsequent membrane delivery would occur on a slower time scale.

### Quantitative analysis of Rab underprenylation in cellular model of CHM

**Figure 3:**
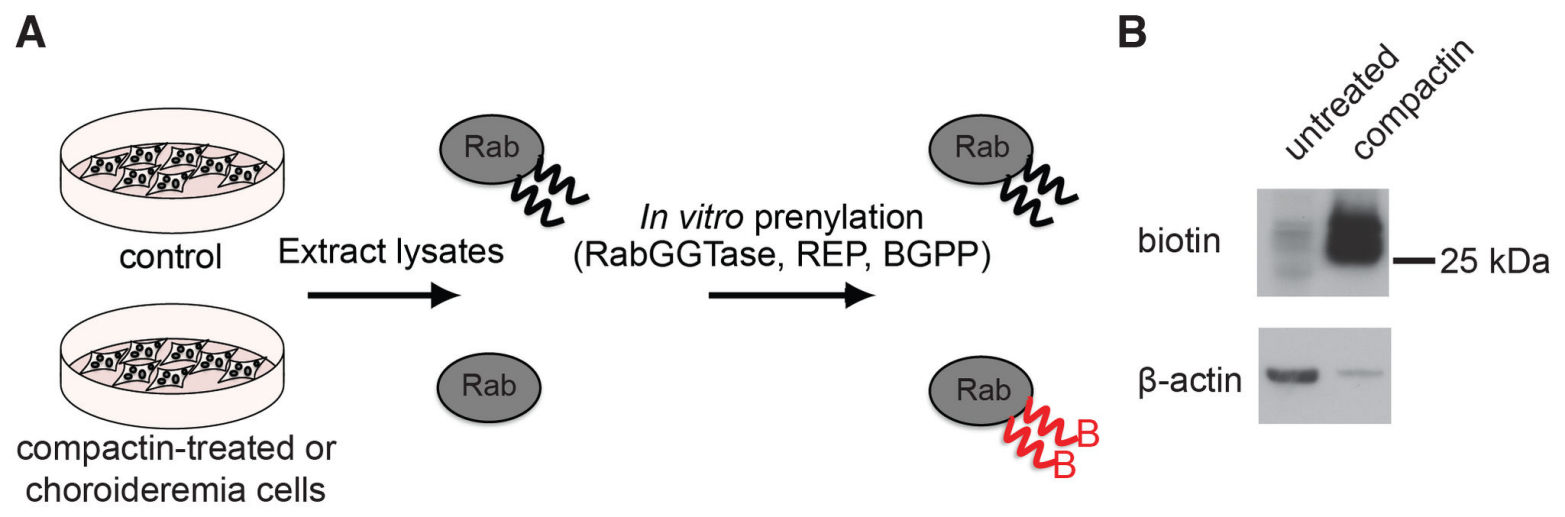
Schematic representation of *in* *vitro* prenylation assay. (A) Cell lysates are subjected to *in* *vitro* prenylation using a recombinant Rab prenylation machinery and the biotin-labeled analogue of GGPP, BGPP. Only those Rabs unprenylated in the cell will be labeled in the assay and can be visualized or purified through the biotin-tag. (B) Western blot analysis of cell lysate from untreated and compactin-treated BHK cells after *in* *vitro* prenylation with BGPP. Unprenylated Rabs are detected via the biotin-tag and actin serves as a loading control. BGPP: biotin-geranyl-pyrophosphate, GGPP: geranylgeranyl-pyrophosphate. Note that compactin treated sample is underloaded and the actual level of unprenylated RabGTPases are higher than they appear from comparison of the band’s intensities.

We previously described an *in vitro* prenylation assay that allows quantification of protein prenylation *in vivo* [12]. In this assay the cells are lysed and subjected to *in vitro* prenylation with exogenously added Rab prenylation machinery and biotin-geranylpyrophosphate (BGPP) as lipid donor (Figure 3). As a result, the *in vivo* unprenylated Rabs are labeled with biotin-geranyl and can be selectively isolated or stained with functionalized streptavidin.

**Figure 4:**
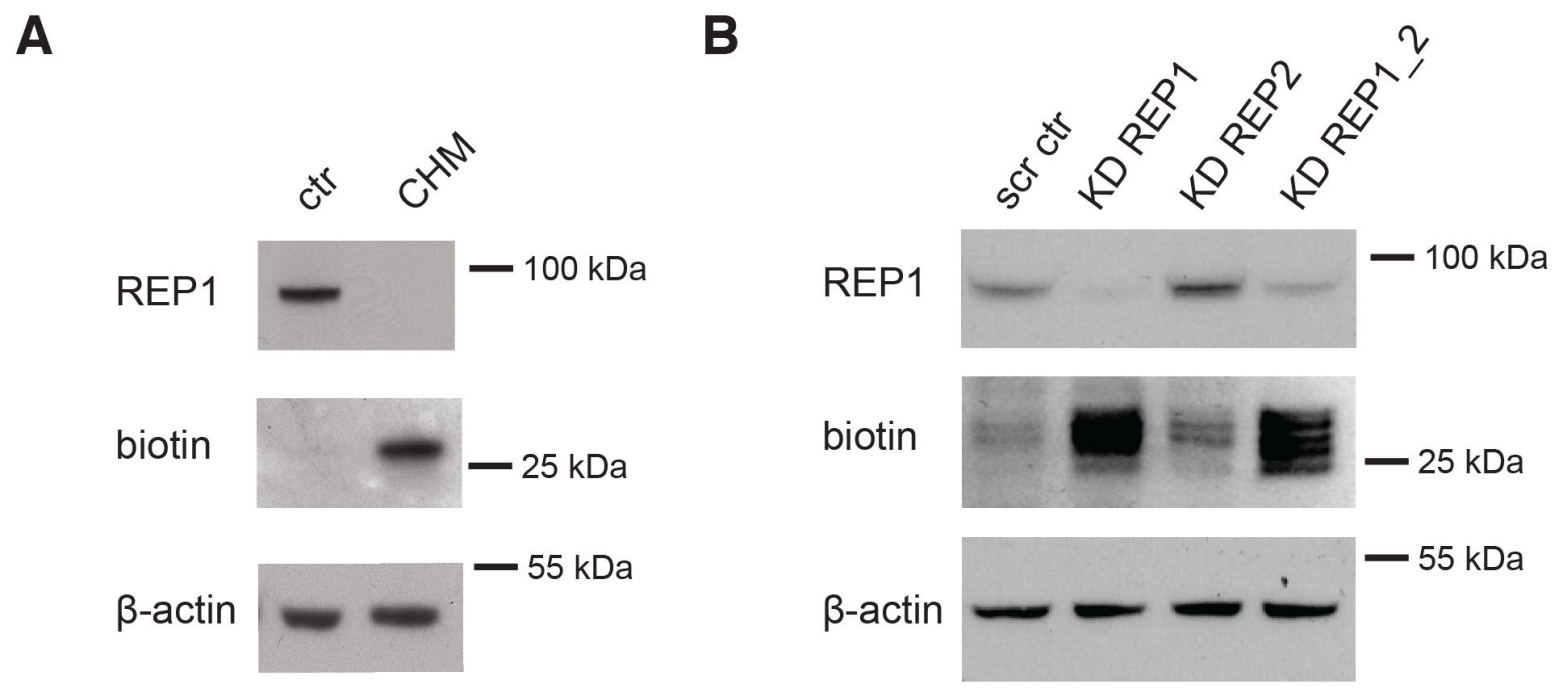
Detection of Rab underprenylation in choroideremia cells and in REP knockdown cells. (A) Lymphoblasts from choroideremia patients (CHM) and control lymphoblasts (ctr) were analyzed for REP1 expression and subjected to *in* *vitro* prenylation with BGPP to detect unprenylated Rabs. (B) Western blot analysis of lysate from HeLa cells expressing shRNAs for REP1 (KD REP1), REP2 (KD REP2) and double knockdown of REP1 and REP2 (KD REP1_2). Cells with scrambled RNA (scr ctr) were used as a control. Cells were analyzed for levels of REP1 expression and levels of unprenylated Rabs by *in* *vitro* prenylation with BGPP. Since no REP2 antibody of quality sufficient for Western blot analysis was available for our studies the levels of REP2 knock down were inferred by Q-PCR analysis of REP2 mRNA in wild type and KD cells (Figure S1).

Using this assay, we were able to detect elevated levels of biotinylated proteins in choroideremia patient cells (Figure 4A). These are most likely to represent Rab proteins that are not sufficiently processed by the remaining REP2 activity and therefore accumulate in an unprenylated state [8]. To verify this assumption experimentally, we generated cell lines with tetracycline-inducible shRNA REP knockdown. The shRNAs were designed to be specific for REP1 or REP2 or for both REPs as described in the materials and methods section and in Table S1. The knockdown of REP1 could be verified by Western blotting but reduction of REP2 expression level could not be ascertained due to the lack of appropriate anti-REP2 antibody. Hence we quantified reduction of REP1 and REP2 mRNA expression using Q-PCR (Figure S1). This analysis demonstrated that expression of both proteins was significantly reduced. The KD cell lines displayed increased levels of unprenylated Rabs especially when both REPs were knocked down (Figure 4B). Interestingly, the effect was much more pronounced when expression of REP1 was reduced than when REP2 was reduced, suggesting that most Rab prenylation occurs with the help of REP1. We also observed that knockdown of REP2 appeared to upregulate the expression of REP-1 (Figure 4B, lane 1 and 3). The significance of this observation is presently unclear. 

### *In vitro* prenylation of Rabs following a block and release assay provides further evidence of Rab prenylation hierarchy

**Figure 5:**
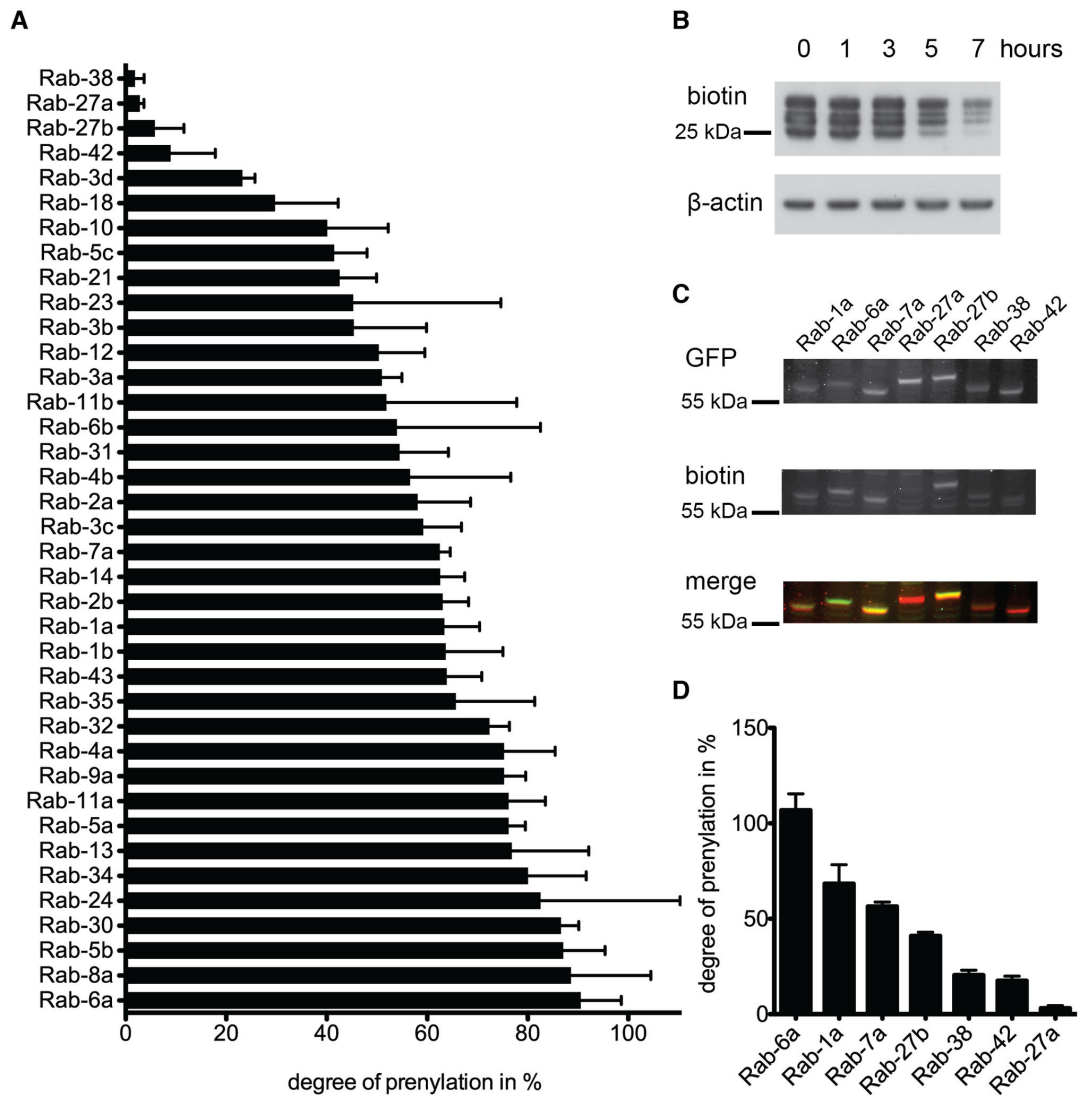
Analysis of Rab prenylation rates *in* *vivo* and *in* *vitro*. (A-B) Analysis of Rab prenylation status after blocking and releasing prenylation *in* *vivo*. HeLa cells were treated with compactin for 24h and then incubated with GGOH for different time periods. Cells were lysed and subjected to *in* *vitro* prenylation with BGPP and recombinant RabGGTase and REP for 6h. (A) Degree of prenylation for each Rab was determined by mass spectrometry. The decrease in signal from the timepoint 0h to 5h was determined by label-free spectral counting and converted into the degree of prenylation for each Rab 5 hours after GGOH addition. The graph represents the mean of three independent experiments (±SEM). (B) Streptavidin-HRP Western blot detection of unprenylated Rabs in the cellular lysates at different timepoints after GGOH addition to compactin-treated cells. The cellular lysates were prenylated with BGPP and RabGGTase as in Figure 3 (C) *In* *vitro* prenylation of lysate from compactin-treated HeLa cells transfected with different GFP-Rabs. *In* *vitro* prenylation reaction was stopped after an hour and subjected to Western blot analysis visualized by infrared Odyssey scanning for total GFP-Rab (GFP/red) and for prenylated biotin-labeled GFP-Rab (biotin/green). Representative blots are shown. (D) The graph represents the percentage of prenylated GFP-Rabs after an hour *in* *vitro* prenylation reaction normalized to complete (overnight) prenylation. Means of three independent experiments are shown (±SEM).

Next we sought to establish whether *in vitro* prenylation with BGPP could be used to determine a prenylation hierarchy of Rabs in the cell. Therefore, we treated HeLa cells with compactin to block prenylation of Rabs [13] and then incubated cells with geranylgeraniol (GGOH) for different time periods. GGOH was shown to convert to GGPP in the cells [14,15] leading to a subsequent rescue of the prenylation block in the absence of *de novo* phosphoisoprenoid synthesis and subsequently restores prenylation of Rabs (Figure 5B and Supporting Results S1). Assuming the existence of a Rab prenylation hierarchy, one would expect Rab proteins with higher affinity for REPs to be prenylated faster than the lower affinity Rabs. To this end, the unprenylated Rabs in the cell lysate were *in vitro* prenylated with BGPP, purified by streptavidin chromatography and analyzed by mass spectrometry (Figure 5A). The relative abundance of Rabs over the time course was determined by the spectral counting label-free method [16-18] . The relative decrease in signal from the timepoint 0 to timepoint 5 was determined and converted into the degree of prenylation for each Rab after 5 hours (Figure S2, Tables S2 and S3 and and Supporting Results S1).This allowed us to determine the extent of *in vivo* prenylation for each of the identified Rabs in the chosen time window (Figure 5B). This analysis revealed that the extent of *in vivo* Rab prenylation varied by more than tenfold. Thus, for the first time we provide the evidence for a Rab prenylation hierarchy in the cell. 

Interestingly, Rab27a, which is expressed in the cell layers that degenerate in choroideremia and has been reported to be underprenylated in choroideremia patient cells [9], displays one of the slowest prenylation rates, which is approximately an order of magnitude slower than that of other Rabs. This confirms that Rab27a is more prone to underprenylation compared to Rabs with a faster prenylation rate. Conversely, Rabs that were previously shown to be prenylated at a faster rate, e.g. Rab1a and Rab7a, were found higher in the Rab prenylation hierarchy (Figure 5A). In addition to Rab27a also Rab38, Rab27b and Rab42 were found near the bottom of the prenylation hierarchy. 

In order to verify the results obtained by the block and release assay, we performed an additional *in vitro* prenylation study. GFP tagged Rabs from the lower, middle and upper end of the prenylation hierarchy were expressed in compactin-treated HeLa cells which results in production of unprenylated proteins. The subsequent *in vitro* prenylation of the lysate revealed differences in the prenylation extent of the different Rabs in accord with our global prenylation analysis (Figure 5C-D). In conclusion, Rab27a is prenylated slowly *in vivo* and *in vitro*, but in addition also other Rabs like Rab38 and Rab42 show slow prenylation kinetics and could therefore potentially play a role in choroideremia.

### Rescue of underprenylation in choroideremia by overexpression of REP1 but not REP2

**Figure 6:**
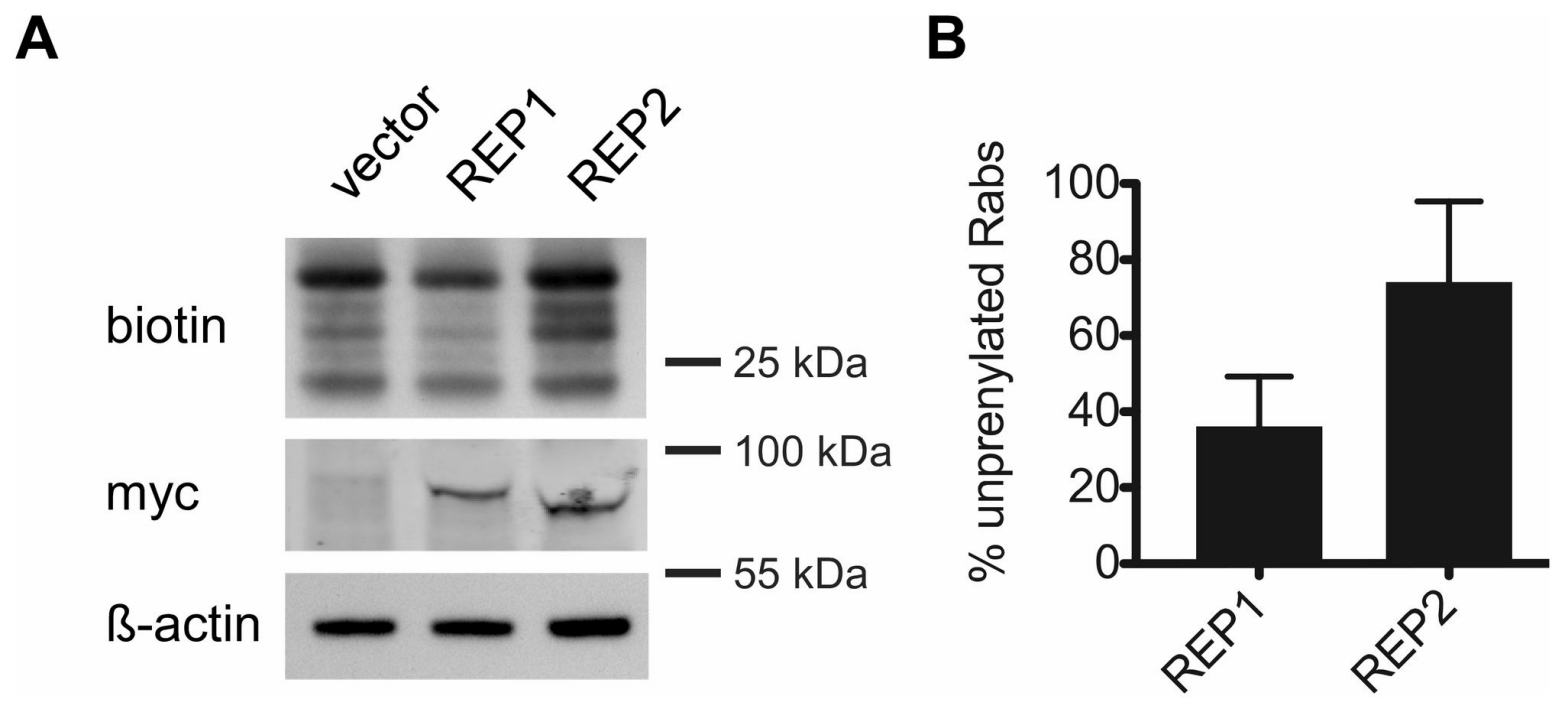
Rescue of underprenylation in REP1 knockdown cells by REP1 but not by REP2. (A) REP1 knockdown cells were transfected with rat REP1-myc, REP2-myc or the vector control. The cell lysate was subjected to the *in* *vitro* prenylation with BGPP to quantitate the levels of unprenylated Rabs. (B) Quantification of unprenylated Rabs in REP1 knockdown cells transfected with REP1 or REP2. The Western Blot signal of the unprenylated Rabs was normalized to the β-actin signal to account for differences in loading. REP1 knockdown cells transfected with vector only were taken as reference representing maximal underprenylation of Rabs in this model and therefore shown as 100%.The graph represents the mean of three independent experiments (± SEM). P-levels are denoted above the bars and were determined with the two-tailed Student’s t-test.

In choroideremia patients, the loss-of–function mutations of REP1 solely lead to the loss of vision. It is hypothesized that REP2 can compensate for the loss of REP1 and maintain physiological levels of prenylated Rabs in all tissues but the eye. Since REP1 and REP2 share 75% amino acid identity, it was suggested that increased levels of REP2 could rescue the lack of REP1 in choroideremia cells [4]. While in the ideal case such experiments should be done in the cell lines derived from CHM patients our efforts to obtain sufficiently high transformation efficiency in immortalized CHM lymphoblast and fibroblast cell lines were unsuccessful (not shown). Hence to investigate the proposed functional redundancy of REP1 and REP2, we made use of previously generated REP1 knockdown cells. To test whether REP2 has the ability to rescue the loss of REP1, we overexpressed REP1 or REP2 in the knockdown cells and subjected the cell lysate to the *in vitro* prenylation assay. The level of unprenylated Rabs in the knockdown control cells (scr ctr), representing newly synthesized Rabs and/or a pool of Rab proteins that is unprenylated under physiological conditions [12], was not significantly different to the REP1 knockdown cells rescued by REP1 overexpression (Figure 6 and Supporting Results S1). However, an elevated level of unprenylated Rabs, comparable to the rescue of the REP1 knockdown with the vector control, could be seen in the rescue with REP2. These findings suggest that despite the high similarity of the two REP isoforms, they fulfill distinct non-redundant functions in the cell. This is direct evidence that REP2 cannot replace REP1 activity in cells with compromised REP1 function even when overexpressed at high levels.

## Discussion

Choroideremia is a rare genetic disease, affecting 4% of the blind population [19]. Patients suffer from retinal degeneration, which results in blindness by the third to fourth decade of their lives. To date, the molecular basis of this disease and the link between genotype and phenotype, is not fully understood. In choroideremia patients three layers in the eye (photoreceptors, retinal pigment epithelium, choroid) are progressively degenerated in the course of the disease. On a molecular level it has been described that loss of REP1 leads to an underprenylation of Rab27a. It has been hypothesized that this results in several defects in vesicle trafficking including transport of disk precursors within the photoreceptor, melanin transport in the retinal pigment epithelium cells or phagocytosis and lysosomal degradation defects of rod outer segment disks [19]. These trafficking defects might be the cause of apoptosis in the cells affected by choroideremia. However, the Rab27a knockout mouse does not appear to have any eye defects [20], suggesting that other Rabs than Rab27a might also contribute to the loss of vision in choroideremia patients.

In this study, we attempt to get insight into Rab prenylation rates within the cells. We provide the evidence that suggests that under the chosen experimental conditions prenylation of Rabs in the cells occurs at different rates. This is in accord with the earlier in vitro data [4,8,9]. We also demonstrate that observed rates of membrane localization are very similar for all tested prenylated Rabs, suggesting that prenylation is the bottleneck for activation and targeting of Rabs. 

In order to investigate the prenylation state of Rabs in the cell, we employed the previously described *in vitro* prenylation assay with a biotin-tagged GGPP analogue [12]. This method is sensitive enough to detect underprenylated Rabs in choroideremia patient cell lines as well as in knockdown cell lines of REP1 and/or REP2. The biotin-tag enables the enrichment and quantification of unprenylated Rabs by mass spectrometry. This allows us to test the idea of Rab prenylation hierarchy *in cells*. Therefore, we performed a block and release assay to determine the prenylation rate of each Rab after complete inhibition of prenylation by a statin compound, compactin. One potential caveat of this approach is the possible effect of underprenylation on the folding of RabGTPases. Although this issue was not examined in this study it is worth noting that RabGTPase are small single subunit biophysically stable proteins that makes large impact of such misfolding unlikely. Here, for the first time, we provide evidence for existence of Rab prenylation hierarchy *in vivo*, in which a subset of Rabs displays a slower prenylation rate and therefore might be more vulnerable to underprenylation in the case of a general decrease of the prenylation capacity. It should be noted that complete inhibition of the mevalonte pathway results in a significant challenge of cellular physiology and the obtained findings need to validated using an independent method. Interestingly, Rab27a shows a low affinity for REP1 and a slow prenylation rate compared to other Rabs and therefore cannot compete favorably with other Rabs for prenylation. Additionally, Rab27a is among the Rabs with the slowest rate of GTP hydrolysis [8]. Therefore newly synthesized Rab27a remains in the GTP-bound form longer than other Rabs, in which it binds REP with low affinity and thus displays low prenylation efficiency. This supports the *in vitro* data, reporting slower prenylation kinetics of Rab27a in comparison to other Rabs. Gordiyenko et al. [21] suggest that underprenylation of multiple Rabs causes the deficiencies seen in choroideremia cells and it is hypothesized that this subset of Rabs preferentially requires REP1 for prenylation. It remains to be further elucidated whether other Rabs that are low in the prenylation hierarchy are also affected by underprenylation in choroidermia cells. Importantly, Rab38, which has a slow *in vivo* prenylation rate, causes an ocular hypopigmentation and thinning of the retinal pigment epithelium when mutated in mice [22,23]. Furthermore, it has been reported that Rab38-deficient rats serve as a model for Hermansky-Pudlak syndrome, a prenylation deficiency disease [24] showing a similar phenotype as seen in humans affected by this disease. In choroideremia patients, Rab38 could remain in an unprenylated and therefore non-functional state, causing similar eye defects observed in the mouse model and thus resulting in impaired vision. To date, four melanosome specific Rabs, Rab27a, Rab27b, and Rab38 have been described [25], all of which showed slow prenylation *in vivo*. Therefore, we suggest that underprenylated Rab27a, Rab27b and Rab38 together may lead to the degeneration of the melanosome enriched RPE. Interestingly, melanosome synthesis in RPE cells is completed before birth, in comparison to melanosome synthesis in melanocytes, which occurs throughout life. Due to the malfunction of melansome-specific Rabs in CHM patients, melanosomes may not be maintained and therefore in tissues where no regeneration of melanosomes takes place, such as the RPE, these cells might undergo apoptosis.

In order to test the specificity of each REP for the pool of unprenylated Rabs, we performed rescue experiments. REP1 knockdown cells with elevated levels of unprenylated Rabs served as models for choroideremia patient cells, resulting in elevated levels of unprenylated Rabs. This enrichment of underprenylated Rabs could be reversed by overexpression of REP1, but not REP2. These results suggest that each REP prenylates a specific pool of Rabs due to a higher affinity or faster turnover rate. These findings are in keeping with the accumulation of unprenylated Rabs despite the presence of REP2 and underline the importance of a REP1-based gene therapy for choroideremia [26-28]. One potential caveat of these experiments is associated with the use of rat REP1 gene to complement the REP1 KD in human cell line. Rat and human REP1 genes share 83% identity with the majority of substitutions concentrated in the unstructured insert region and the C-terminus of the molecule. The fact that expression rat REP1 efficiently rescues the KD of endogenous REP1 in HeLa cells suggests that it is fully functional in this cellular model.

A recent publication describes trafficking defects in peripheral cells of choroideremia patients, which also seems to be related to the loss of REP1 [29]. However, blindness is the only symptom occurring in patients. In all other tissues but the eye, REP2 can compensate for the loss of REP1 in order to generate physiological levels of prenylated Rabs. Whether this is due to the composition of Rabs in this specific tissue or the expression level of REPs, or a combination of both, needs to be further studied in order to fully elucidate the complex interplay of factors controlling Rab prenylation.

## Materials and Methods

### Expression constructs, protein expression and purification

CFP-Rab1a, YFP-Rab7a, Citrine-Rab7a and Cherry-Rab27a were expressed in *E. coli* and purified as described [30]. RabGGTase α and β were cotransformed in *E. coli* and expressed and purified likewise [30,31]. Expression of rat REP1 in SF21 cells and subsequent purification was performed as described [30,31]. 

The vectors for expression of rat REP1, human REP2 and rat RabGGTase in mammalian cells were constructed as follows: the pPRIG vector was obtained from Dr Pognonec, (http://www.unice.fr/LPCM/PRIG/PRIG_home.html tinyurl http://tiny.cc/prlG4 [32], [33]. REP1 and REP2 were PCR-amplified using primers that introduced a 5’ *Cla*I site and a 3’ *Not*I site. The PCR product was digested with *Cla*I and *Not*I and cloned into the pPRIG vector pre-digested with the same enzymes. The myc-tagged REP vectors were generated by PCR amplification of rat REP1 and human REP2 with overhangs containing the myc-tag and restriction sites for *Nhe*I/*Not*I and subcloned into the pcDNA3.1(-) (Invitrogen) with the same restriction sites. Previous biochemical and structural analysis demonstrated that C-terminus is not involved in the interaction with RabGGTase or RabGTPases [4] and references therein. 

The vectors for knockdown of REP1 and/or REP2 and scrambled control [34] were constructed by inserting double synthetic oligonucleotides at the *Hind*III/*Bgl*II site of the pTER+ plasmid [35] (see supplementary information for sequences). Knockdown constructs for REP1 and/or REP2 were designed with sequence specificity for human REP.

### *In vitro* prenylation of recombinant protein

Citrine-Rab7a and Citrine Rab27a were prenylated *in vitro* and purified as a complex with REP1 as described previously [36].

### MALDI-TOF Analysis of Recombinant RabGTPases

Protein samples were desalted using small GF spin columns DYEx2.0 Spin Kit, (Qiagen, Germany) and mixed with an equal volume of matrix (saturated sinapinic acid solution in 0.1% TFA, 50% acetonitrile). The mixture was spotted onto a MALDI sample plate and air-dryed. Spectra were recorded on a Voyager DE Pro Biospectrometry workstation from Applied Biosystems (Weiterstadt Germany) with the following settings: acceleration voltage = 25 kV, grid voltage = 93 %, extraction delay time = 750 ns and guide wire = 0.3 %. The laser intensity was manually adjusted during the measurements in order to obtain optimal signal to noise ratios. Spectra recording and data evaluation was performed using the supplied Voyager software package.

### Cell culture

All cells were maintained at 37°C in 5% CO_2_. A431 cells were maintained in DMEM (Gibco) with 4.5 g/L glucose and L-glutamine, supplemented with 10% fetal bovine serum (FBS, Gibco), 1% penicillin/streptavidin (Gibco) and with an additional 1.5 g/l NaHCO_3_ and were split every 2-3 days. CHM2100 cells (Lonza), Epstein Barr transformed B-lymphocytes from a choroideremia patient, and IM-9 lymphoblasts (ATCC) were maintained in RPMI medium (Gibco) including L-glutamine and were supplemented with 10% FBS. HeLa cells were obtained from Sigma Aldrich and cultured in DMEM supplemented with 10% FBS.

Where stated, cells were treated with 5 µM compactin or, in untreated cells, with DMSO at 1/1000 v/v. For the inducible knockdown, 1 µg/ml doxycycline was added and cells were harvested 72h after doxycycline induction.

### Transfection

A431 cells were electroporated according to the protocol provided by Amaxa. HeLa cells were transfected with FuGene HD (Roche) according to the manufacturer’s protocol. For selection of stable knockdown cells, cotransfection of the pTER knockdown constructs and pcDNA6/TR (Invitrogen) was performed; antibiotics selection with 200 µg/ml zeocin (Invitrogen) and 10 µg/ml blasticidin (Astral Scientific) was started 24 h after transfection.

### Microinjection

A431 cells were microinjected using an Eppendorf micromanipulator and transjector, on a Zeiss inverted microscope enclosed in an Okolab temperature controlled cage. Cells were injected and photographed in imaging medium (150mM NaCl, 5mM KCl, 1mM MgCl_2_, 1mM CaCl_2_, 20mM Hepes, 10mM Glucose) at 37°C in 5% CO_2_ and were otherwise incubated in growth medium as for culturing. Cells were injected with 40 µM of recombinant protein and imaged for 24 hours using a fluorescent inverted microscope. In a typical experiment 15 cells were microinjected and imaged for each Rab GTPase. 

### Microscopy

A431 cells were imaged at different timepoints after microinjection of RabGTPase proteins with a Leica TCS-SP5 microscope equipped with a 37°C incubator. Images were acquired with 40x and 63x oil immersion objectives. The different fluorophores were detected with the following settings: CFP excitation at 405 nm, CFP emission at 470-500 nm; YFP excitation at 514 nm, YFP emission at 530-600; mCherry excitation at 561 nm, mCherry emission at 580-670 nm. Image analysis and processing was carried out using ImageJ.

### *In vitro* prenylation and pull-down of biotin-tagged proteins

Cells lysates were produced as described in Nguyen et al., 2009. In brief, cells were washed with PBS and incubated with prenylation buffer (50 mM Hepes pH 7.2, 50 mM NaCl, 20 mM MgCl_2_, 5mM DTT and protease inhibitor cocktail (Roche)) for 15 min at 4°C. Adherent cells were then scraped and passed through a 27 gauge needle 20 times, followed by centrifugation at 1,500xg for 5 min at 4°C. The supernatant was transferred to a fresh tube and centrifuged at 100,000xg for 1 h at 4°C. The supernatant was used for the *in vitro* prenylation reaction. The prenylation machinery (2 μM REP1 and 2 μM RabGGTase) and a GGPP analogue (5 μM Biotin-GPP) was then added and incubated at room temperature for 4-6 h. The prenylation reaction was ended with sample buffer and resolved by SDS-PAGE followed by Western blotting and detection with streptavidin-HRP using film. The intensities of the bands were quantitatively analyzed using ImageJ software.

For mass spectrometric analysis the cell lysate from five 15 cm tissue culture dishes was prepared and subjected to the *in vitro* prenylation assay. The prenylated lysate was alkylated with 15 mM iodoacetamide for 30 min at room temperature in the dark. Then the lysate was incubated with magnetic streptavidin beads (NEB) for 1 h at room temperature, followed by washes with 1% NP-40, 4 M urea, 4 M guanidine hydrochloride and 40 mM NH_4_HCO_3_ three times each for 10 min at 4°C.

### Tryptic Digest and Mass Spectrometry

Streptavidin beads with bound reduced and alkylated proteins (from the *in vitro* prenylation and pull-down of biotin-tagged proteins) were transferred into 10% acetonitrile, 40 mM NH_4_HCO_3_ and digested with 1 µg of modified sequencing grade trypsin, bovine pancreas (Roche) for 2 hours at 37°C, 700 rpm on a Thermomixer Comfort (Eppendorf); followed by the addition of a further 1 µg trypsin and incubation for 6 hours at 37°C, 700 rpm. The recovered digest liquor was dried down and made up to 25 µL with 1% formic acid, 2% acetonitrile.

### Capillary (Cap) HPLC-LTQ-Orbitrap

Acidified digests were subjected to CapHPLC-MS/MS analysis using a Prominence nano HPLC system (Shiumadsu, Kyoto, Japan) interfaced with a linear ion-trap (LTQ)- Orbitrap Velos hybrid mass spectrometer (Thermo Fischer Scientific, Bremen, Germany). Digests were loaded onto a 300 Å, 300 μm × 5 mm C18 trap column (Dionex Acclaim® PepMap™ μ-Precolumn) at 30 µL/min in 100% solvent A (0.1% (v/v) aqueous formic acid) for 3.5 minutes at 40 °C and subsequently back-flushed onto a pre-equilibrated analytical column (Vydac Everest C18 300 Å, 150 μm × 150 mm, Alltech) using a flow rate of 1 µL/min and 98% solvent A, 2% solvent B (80% (v/v) ACN / 20% (v/v) H_2_O, containing 0.1% (v/v) formic acid). Peptides were separated at 40 °C using a sequence of linear gradients: to 45% B over 72.5 minutes; to 75% B over 15 minutes; and, to 95% B over 5 minutes and then holding the column at 95% B for 10 minutes. Eluate from the analytical column was introduced into the LTQ-Orbitrap Velos throughout the entire run via a Nanospray Flex Ion Source (Thermo Fisher Scientific) containing a 30 μm inner diameter uncoated silica emitter (New Objective). Typical spray voltage was 1.5 kV with no sheath, sweep or auxiliary gases used. The heated capillary temperature was set to 250°C. The LTQ- Orbitrap Velos was controlled using Xcalibur 2.2 software (Thermo Fisher Scientific) and operated in a data-dependent acquisition mode to automatically switch between Orbitrap-MS and ion trap-MS/MS. The survey full scan mass spectra (from m/z 380 - 1700) were acquired in the Orbitrap with a resolving power set to 30,000 (at 400 m/z) after accumulating ions to an automatic gain control (AGC) target value of 1.0 x 10^6^ charges in the LTQ. MS/MS spectra were concurrently acquired on the 20 most intense ions from the survey scan in the LTQ filled to an AGC target value of 3.0 x 10^4^. Charge state filtering, where unassigned precursor ions were not selected for fragmentation, and dynamic exclusion (repeat count 1, repeat duration 30 s, exclusion list size 500, exclusion duration 90 s) were used. Fragmentation conditions in the LTQ were: 35% normalized collision energy; activation q of 0.25; 10 ms activation time; and, minimum ion selection intensity 5000 counts. Maximum ion injection times were 500 ms for survey full scans and 10 ms for MS/MS. 

### Data Processing

Tandem mass spectra were processed using Proteome Discoverer (version 1.3, Thermo Fisher Scientific) and submitted to Mascot (version 2.2.06, Matrix Science). Variable modifications: carbamidomethyl-cysteine, deamidation (asparagine, glutamine); oxidation (methionine). Enzyme: trypsin, 2 missed cleavages, MS tolerance 20ppm, MSMS tolerance 0.8 Da. Scaffold (Proteome Software, Portland, Oregon, USA) [37] was used to validate Mascot protein identifications.  Scaffold probabilistically validates these peptide identifications using PeptideProphet (3) and derives corresponding protein probabilities using ProteinProphet (2). The raw spectral counts and their normalization are summarized in the Tables S2 andS3 respectively.

### Label-free spectral counting

The relative quantification of proteins detected by mass spectrometry was determined by spectral counting of unweighted spectral counts. The results were filtered for ≥ 2 peptides and ≥ 5 spectra per identified protein and normalized to account for variations between the runs. In brief, the spectral counts were normalized by dividing the spectral counts of each protein by the total spectral counts of the same sample and multiplied by the total spectral counts in the control sample [38]. For the block and release assay the relative decrease in signal from timepoint 0 hours, which reflects the total pool of unprenylated Rabs, to the timepoint 5 hours was converted into the degree of prenylation for each identified Rab 5 hours after GGOH addition. See Table S2 for mass spectrometry analysis.

### *In vitro* prenylation kinetics

HeLa cells were transfected with pEGFP-C1-Rab constructs and 12 h post-transfection subjected to compactin treatment for 24 h. Lysates were prepared as described above and subjected to *in vitro* prenylation for 1 h and overnight. The ratio of prenylated GFP-Rabs after 1 h prenylation was determined by quantitative Western blot analysis with the infrared Odyssey system and normalized to the fully prenylated samples, which were incubated overnight.

### REP1 and REP2 rescue in knockdown cells

Stable REP knockdown or scrambled control cells were treated with doxycycline (1 µg/ml) for 72h. Transfection with pcDNA REP1 or REP2 constructs was performed 36 h prior to harvesting the cells. The cells were harvested and lysate prepared according to the protocol given above. To remove REP the lysate was fractionated using 50 kDa Amicon centrifugal filters and washed three times with prenylation buffer. The flow-through was concentrated and subjected to *in vitro* prenylation.

### RT-PCR quantification

Cells were harvested 72 h after doxycycline addition (1 µg/ml), RNA extraction and cDNA synthesis (CellSure, Bioline) were performed according to the manufacturer’s protocol. The real time PCR reaction consisted of cDNA template (diluted 1:20), forward and reverse primers (200 nM final concentration), and Platinum SYBR Green qPCR Supermix-UDG (Invitrogen) in a total volume of 20 µl. Glyceraldehyde 3-phosphate dehydrogenase (GAPDH) was used as normalization reference. Quantitative real time PCR was carried out in triplicate on three independent templates on a 7500 Real-Time PCR System (Applied Biosystems). For analysis of the Ct values the ΔΔCt method was applied. To test for contamination standard control PCR reactions were performed. 

## Supporting Information

Figure S1 **Quantitative RT-PCR analysis of REP mRNA expression.** Real-time analysis of HeLa cells expressing shRNAs for REP1 (KD REP1), REP2 (KD REP2) and shRNA with dual-specificity for REP1 and REP2 (KD REP1_2). Cells expressing scrambled RNA (scr ctr) were used as a control. Knockdown efficiency was determined by quantitative real-time PCR. Scrambled RNA cells served as a control for the expression levels of (A) REP1 and (B) REP2. The shRNA expression was induced by doxycycline treatment for 72h. Values given are means (±SEM) (n=3).
(TIF)

Figure S2 **Identification of Rab GTPases from compactin treated HeLa cells by mass spectrometry.** Compactin treated HeLa lysate was prenylated *in* *vitro* with BGPP. Biotin-geranyl tagged Rab proteins were enriched by pull-down and subjected to mass spectrometry analysis. Identified Rabs are shown with their number of unweighted spectral counts. The graph represents means (±SEM) of three independent experiments.
(TIF)

Results S1 (DOCX)

Table S1 **Oligo sequences for shRNA REP knockdown construct and scrambled RNA control.**
(DOCX)

Table S2 **Raw mass spectrometry data of the time resolved in vitro prenylation experiment.**
(XLS)

Table S3 **Normalization and quantification of the time resolved in vitro prenylation experiment data.**
(XLSX)
