# Supplementary figures and images for: Rab GTPase Prenylation Hierarchy and Its Potential Role in Choroideremia Disease
Source: PLoS One. 2013 Dec 16;8(12):e81758. doi: 10.1371/journal.pone.0081758 (PMC3864799; doi:10.1371/journal.pone.0081758)

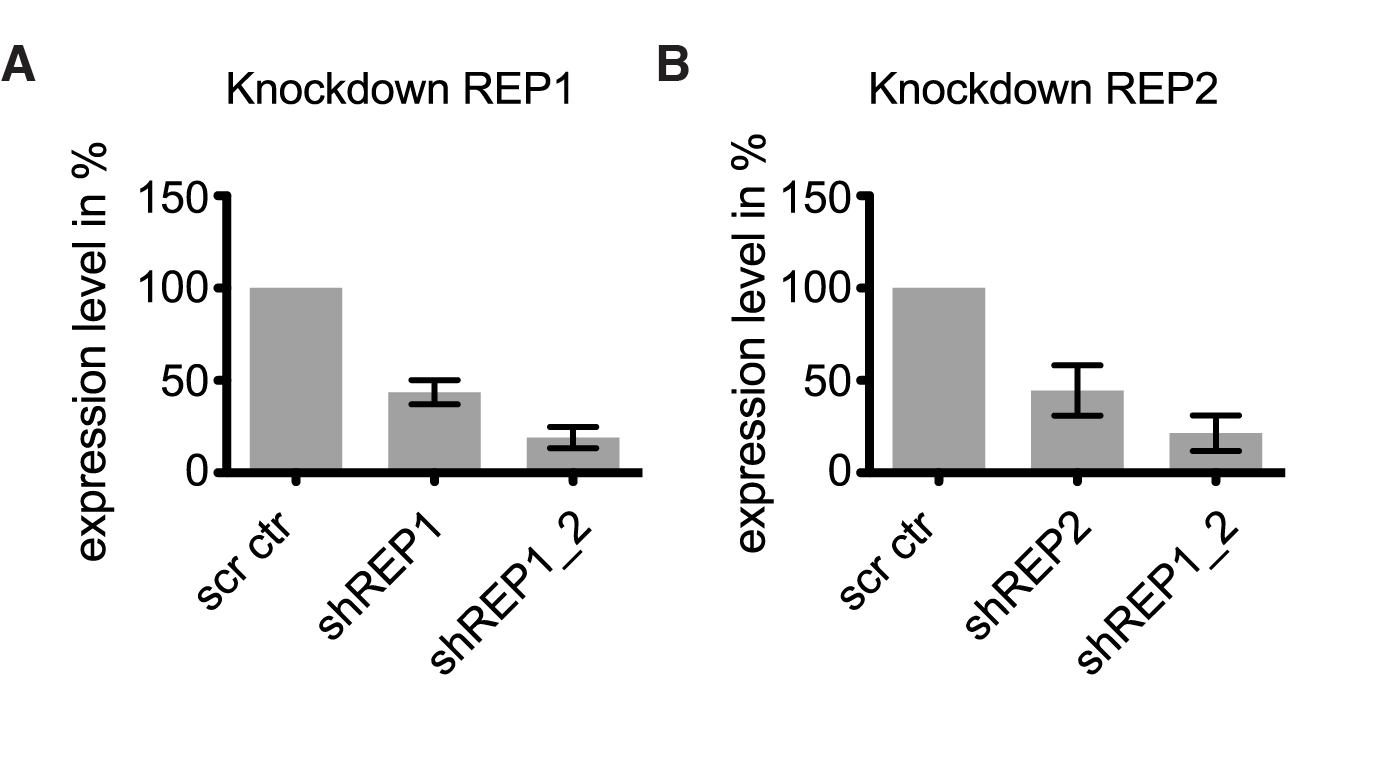

Supplement: Figure S1 — Quantitative RT-PCR analysis of REP mRNA expression. Real-time analysis of HeLa cells expressing shRNAs for REP1 (KD REP1), REP2 (KD REP2) and shRNA with dual-specificity for REP1 and REP2 (KD REP1_2). Cells expressing scrambled RNA (scr ctr) were used as a control. Knockdown efficiency was determined by quantitative real-time PCR. Scrambled RNA cells served as a control for the expression levels of (A) REP1 and (B) REP2. The shRNA expression was induced by doxycycline treatment for 72h. Values given are means (±SEM) (n=3). (TIF) [file pone.0081758.s001.tif]

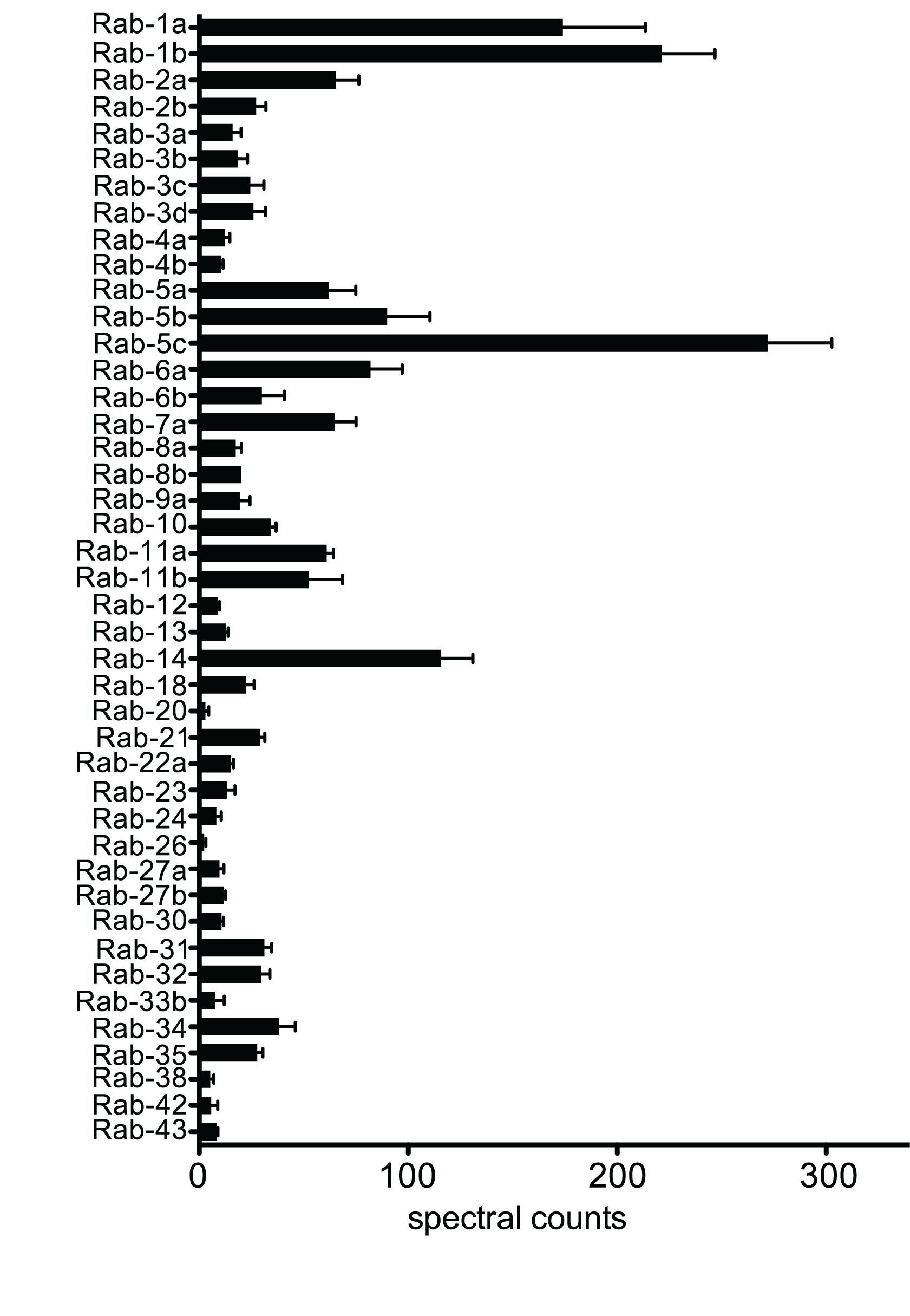

Supplement: Figure S2 — Identification of Rab GTPases from compactin treated HeLa cells by mass spectrometry. Compactin treated HeLa lysate was prenylated in vitro with BGPP. Biotin-geranyl tagged Rab proteins were enriched by pull-down and subjected to mass spectrometry analysis. Identified Rabs are shown with their number of unweighted spectral counts. The graph represents means (±SEM) of three independent experiments. (TIF) [file pone.0081758.s002.tif]
