# Supplementary material for: Rab GTPase Prenylation Hierarchy and Its Potential Role in Choroideremia Disease
Source: PLoS One. 2013 Dec 16;8(12):e81758. doi: 10.1371/journal.pone.0081758 (PMC3864799; doi:10.1371/journal.pone.0081758)
Supplement: Results S1 — (DOCX) [file pone.0081758.s003.docx]

**Supporting Results**

**Analysis of REP knockdown**

To determine the efficiency of the REP1 knockdown in HeLa cells, we performed RT-PCR analysis (Fig. S1) and Western blot analysis (Fig. 4). The mRNA expression levels of REP1 and REP2 were determined by quantitative real-time PCR. These showed a knockdown efficiency of REP1 of around 60% for the REP1 knockdown cell line and of around 80% for the REP1_2 knockdown cell line (Fig. S1A). The REP2 knockdown led to a REP2 knockdown of around 55% in the REP2 knockdown cell line and around 80% in the REP1_2 knockdown cell line (Fig. S1B). The shRNA expression leads to a reduction of REP1 mRNA levels to about 40%. We could confirm this result by Western blot analysis of REP1 protein levels. Due to the lack of a commercially available REP2 antibody, the knockdown of REP2 could not be confirmed on the protein level. Additionally, the knockdown of REP1 correlates with an increase in unprenylated Rabs as observed by the *in vitro* prenylation assay with BGPP (Fig.4).

**Identification of endogenous RabGTPases by mass spectrometry analysis**

Rabs that are unprenylated in the cell can be labeled with a biotin-tag using the *in vitro* prenylation assay, thus allowing enrichment and purification of Rabs for further analysis. *In vitro* prenylation of compactin treated HeLa lysate with BGPP, followed by enrichment with magnetic streptavidin beads and mass spectrometry analysis resulted in the identification of 43 Rabs by comparing the spectra with the sequence databases (Fig. S2).

**Specificity of Rab prenylation by REP1 or REP2**

In order to test if REP1 and REP2 show preferential prenylation of certain Rabs, we performed an *in vitro* prenylation assay with either REP1 or REP2 and compared the prenylation efficiency for individual Rabs by mass spectrometry analysis. First we performed a prenylation timecourse of *in vitro* prenylation of lysate from compactin treated HeLa cells with BGPP with either human REP1 or REP2 (Fig. S3B). The intensity of labeling of unprenylated Rabs with BGPP showed no difference between the prenylation kinetics of REP1 and REP2. The prenylation reaction was saturated after 1 to 2 hours of *in vitro* prenylation. Therefore, we chose to further analyze the 20 minute timepoint with mass spectrometry analysis to reveal differences in the prenylation efficiency of REP1 and REP2 of individual Rabs. Lysate from compactin treated cells was subjected to *in vitro* prenylation with BGPP and either REP1 or REP2 and the reaction was stopped after 20 minutes. Subsequently, pull down with magnetic streptavidin beads and mass spectrometry analysis was performed. The spectral counts for individual Rabs were analyzed and compared for the reaction with REP1 and REP2 (Fig. 3 A). The spectral counts reflect on the one hand the relative protein abundance, and on the other hand the *in vitro* prenylation efficiency. Since the prenylation reaction was carried out with the same lysate for both conditions, differences in protein abundances can be ruled out. In addition, the spectral count analysis did not reveal any significant differences between the prenylation with REP1 and REP2. Taking into account that the *in vitro* prenylation reaction is carried out at 2 µM REP, it exceeds the endogenous REP concentrations by orders of magnitudes. Therefore, it is possible that differences in the prenylation efficiencies between REP1 and REP2, which are due to differences in affinities for certain Rabs, are overcome in this experimental setting, which leads to similar results for both REP isoforms. Unfortunately, the sensitivity of this method does not allow detection of individual Rabs with the *in vitro* prenylation reaction at endogenous REP concentrations. Therefore, we cannot conclude from this experiment if individual Rabs are preferentially prenylated by one of the REP isoforms at endogenous REP concentrations.
