# Supplementary material for: Rab GTPase Prenylation Hierarchy and Its Potential Role in Choroideremia Disease
Source: PLoS One. 2013 Dec 16;8(12):e81758. doi: 10.1371/journal.pone.0081758 (PMC3864799; doi:10.1371/journal.pone.0081758)
Supplement: Table S1 — Oligo sequences for shRNA REP knockdown construct and scrambled RNA control. (DOCX) [file pone.0081758.s004.docx]

**Table S1. Oligo sequences for shRNA REP knockdown construct and scrambled RNA control.**

| Sense oligo REP1 | 5’ gatcccgcatgaagatgtcgaagagttcaagagattcttcgacatcttcatgctttttggaaa 3’ |
| --- | --- |
| Antisense oligo REP1 | 5’ agcttttccaaaaagcatgaagatgtcgaagaatctcttgaactcttcgacatcttcatgcgg 3’ |
| Sense oligo REP2 | 5’ gatcccgctcaaatgtgcagtatagttcaagagattatactgcacatttgagctttttggaaa 3’ |
| Antisense oligo REP2 | 5’ agcttttccaaaaagctcaaatgtgcagtataatctcttgaactatactgcacatttgagcgg 3’ |
| Sense oligo REP1_2 | 5’ gatccccagctgcatgttcaagagttcaagagattcttgaacatgcagctgcttttttggaaa 3’ |
| Antisense oligo REP1_2 | 5’ agcttttccaaaaaagcagctgcatgttcaagaatctcttgaactcttgaacatgcagctggg 3’ |
| Sense oligo scrambled | 5’ gatcccggcctaaggttaagtcgccctcgctcgagcgagggcgacttaaccttaggtttttggaaa 3’ |
| Antisense oligo scrambled | 5’agcttttccaaaaacctaaggttaagtcgccctcgctcgagcgagggcgacttaaccttaggccgg3’ |
